# Cognitive, Behavioral and Emotional Empathy in Pharmacy Students: Targeting Programs for Curriculum Modification

**DOI:** 10.3389/fphar.2016.00096

**Published:** 2016-04-19

**Authors:** Cassandra A. Tamayo, Mireille N. Rizkalla, Kyle K. Henderson

**Affiliations:** ^1^Chicago College of Osteopathic Medicine, Midwestern University, Downers GroveIL, USA; ^2^Department of Clinical Integration, Chicago College of Osteopathic Medicine, Midwestern University, Downers GroveIL, USA; ^3^Department of Physiology and Department of Osteopathic Manipulative Medicine, Chicago College of Osteopathic Medicine, Midwestern University, Downers GroveIL, USA

**Keywords:** education, pharmacy, empathy, behavioral empathy, emotional empathy, cognitive empathy, interdisciplinary, Jefferson scale of medical student empathy

## Abstract

**Introduction:** Empathy is an essential trait for pharmacists and is recognized as a core competency that can be developed in the classroom. There is a growing body of data regarding levels of empathy in pharmacy students; however, these studies have not measured differences in behavioral, cognitive, and emotional empathy. The goal of this study was to parse the underlying components of empathy and correlate them to psychosocial attributes, with the overall goal of identifying curriculum modifications to enhance levels of empathy in pharmacy students.

**Methods:** IRB approval was obtained to measure empathy levels in pharmacy students attending Midwestern University. An online, anonymous survey administered through a secure website (REDCap) was used. This survey utilized the Jefferson Scale of Empathy (Medical Student version) and included questions regarding demographics and personality traits. Empathy questions were sub-divided into behavioral, cognitive, and emotional categories. Data are presented as mean ± SEM with significance set at P ≤ 0.05.

**Results:** Three hundred and four pharmacy students at Midwestern University participated in a fall survey with an overall response rate of 37%. The average empathy score was 110.4 ± 0.8 on a scale of 20–140; which is comparable to empathy scores found by Fjortoft et al. (2011) and Van Winkle et al. (2012b). Validating prior research, females scored significantly higher than males in empathy as well as behavioral, cognitive, and emotional subcomponents. For the entire population, emotional empathy was significantly higher than cognitive and behavioral empathy (P < 0.05). Furthermore, negative correlations to empathy were observed for self-serving behavior (R D 0.490, P < 0.001), medical authoritarianism (R D 0.428, P < 0.001), and experience of coercion (R D 0.344, P < 0.001).

**Conclusion:** Overall, empathy levels in pharmacy students are similar to prior studies with females scoring higher than males. Emotional empathy may play a greater role than cognitive and behavioral empathy in this group of students. Targeted programs that promote volunteerism and activities that foster responsiveness to patient needs may attenuate self-serving behavior and medical authoritarianism and, therefore, improve empathy levels in pharmacy students.

## Introduction

Pharmacy is a human service profession with a unique position in providing health and functionality to patients. As with all humanistic professions, empathy is an essential trait for pharmacists and is recognized as a core competency that should be developed throughout graduate school. Consistent with this notion, the Accreditation Council for Pharmacy Education mandates that humanistic values and empathy be enriched and assessed as a core educational activity in graduate pharmacy education (Education, 2015).

Empathy is a multidimensional construct with behavioral, cognitive, and emotional domains (Larson and Yao, 2005). Cognitive empathy describes an individual’s capacity to understand another person’s perspective (Fjortoft et al., 2011; Shamay-Tsoory, 2011) as opposed to being self-oriented (Kohut, 1969; Basch, 1983; Eisenberg and Miller, 1987). Emotive empathy describes an affective characteristic, which involves experiencing and internalizing the feelings experienced by others (Eisenberg, 1989; Nunes et al., 2011). Behavioral empathy is action-oriented; it involves the outward expression of internally experienced (cognitive and emotive) processes which can be directed at improving clinical outcomes (Larson and Yao, 2005).

Prior research has sought to delineate the relative contribution between each of these empathy domains on clinical skills. One perspective is that cognitive empathy is the most predominant type of empathy in the medical setting (Halpern, 2003; Hojat, 2009); while others highlight the importance of both the behavioral and emotive aspects (Benbassat and Baumal, 2004; Manolakis et al., 2011). Yet it is also plausible that empathy, in its totality as a trinity, is the ideal vehicle that allows health care providers to practice patient-centered care, in which the patient’s body, mind, and spirit can be evaluated comprehensively. Neuroscientists (Haas et al., 2015), empathy researchers (Hojat et al., 2009), and pharmacy educators (Maine and Vogt, 2009; Vogt and Finley, 2009; Education, 2015) continue to identify a significant need to examine empathy with greater scientific rigor.

The subcomponents of empathy originate at the neural level. Cognitive empathy is an executive function that recruits higher-order brain regions in the prefrontal and temporal cortices (Frith and Singer, 2008; Van Overwalle and Baetens, 2009) that enable “perspective taking,” a process of materializing another’s thoughts and intentions, known as the “Theory of Mind” (Baron-Cohen, 2009). In contrast, emotional empathy is a primitive function that recruits brain regions in the inferior frontal and parietal cortex (Shamay-Tsoory, 2011). This network, collectively known as the mirror neuron system, is instinctive and involved in emotional recognition (Shamay-Tsoory et al., 2009; Shamay-Tsoory, 2011). Behavioral empathy is a construct that is defined as actions taken in response to the internal experience of cognitive and/or emotional empathy. Although behavioral empathy may be triggered by both cognitive and emotional processes (Shamay-Tsoory, 2011), the exact trigger must be distinguished, as they lead to very different clinical behaviors (Nightingale et al., 1991). For example, a cognitively empathetic pharmacist would be more inclined to act upon the content and quality of patients’ symptoms, whereas an emotionally empathetic pharmacist would be more inclined to sympathetically respond to patients’ feelings of pain and suffering.

The current project grew out of a theoretical model that recognizes cognitive empathy as an adaptive function that can be taught. For example, by increasing ‘perspective taking,’ a pharmacist is better equipped to predict motives and health/risk behaviors such as medication compliance and substance abuse (Darbishire et al., 2012). Therefore, cognitive empathy emphasizes the appropriateness of the biopsychosocial model to health care, wherein the full realization of health is achieved within the context of a complex interaction of biological, psychological, and social factors. Theoretical models have hypothesized a linear relationship between cognitive empathy and positive outcomes, meaning that the outcomes progressively improve as a function of an increase in cognitive empathy. In contrast, excessive emotional empathy can cloud the neutrality that is necessary in clinical practice, thus cultivating compassion fatigue, exhaustion, and vicarious traumatization (Linley and Joseph, 2007). The relationship between emotional empathy and clinical outcomes is characterized by a bell-shaped curve, meaning that emotional empathy can be beneficial to a limited extent, but then becomes detrimental in excess (Hojat et al., 2009).

Empathy levels in health related professions can be measured by: self-assessment (first person assessment), patient-rating (second person assessment), and observation (third person assessment) (Hemmerdinger et al., 2007). When large sample sizes are evaluated, self-assessment questionnaires are the most efficient and the Jefferson Scale of Physician Empathy (Hojat et al., 2001), and student empathy (Hojat et al., 2004) were developed for this purpose. Prior research has assessed empathy levels and tracked its temporal and individual psychosocial differences in medical (Mehrabian et al., 1988), paramedic (Nunes et al., 2011) and allied health professionals (Williams et al., 2014), and similar research on pharmacy students is rapidly growing (Vogt and Finley, 2009; Fjortoft et al., 2011; Manolakis et al., 2011; Nunes et al., 2011; Darbishire et al., 2012; Van Winkle et al., 2012a,b, 2013a; Wilson et al., 2012; Chen et al., 2015; Jeon and Cho, 2015; Kerr et al., 2015; Lor et al., 2015). However, very little is known about individual differences in emotional, cognitive, and behavioral empathy. Consequently, sufficient attention has not been directed toward the malleability and enhancement of the subcomponents of empathy in pharmacists-in-training. The goal of this study was to parse the components of empathy and correlate them to psychosocial attributes, thereby elucidating the composition of empathy in pharmacy students and advancing our understanding of what traits promote or erode empathy. A better conceptualization of empathy will not only provide an innovative framework for studying empathy in pharmacy students, but will also help to identify curriculum modifications to optimize empathy subcomponents.

## Materials and Methods

Midwestern University Institutional Review Board (IRB) approval was obtained to measure empathy levels in pharmacy students attending Midwestern University. A voluntary, online, anonymous survey was administered through a secure web-based application called REDCap (Research Electronic Data Capture) hosted at Midwestern University (Harris et al., 2009). This survey utilized the Jefferson Scale of Empathy (Medical Student version). These questions were then sub-divided into behavioral (action), cognitive (perspective-taking), and emotional (expressed feeling), categories and the responses were tabulated (Appendix). Questions targeting personality traits utilized the same seven point Likert scale used by the Jefferson empathy survey. Data was organized with Microsoft Excel and statistical significance determined with Sigma Stat 12.5 software. For group comparisons, normality and variance were tested and appropriate Analysis of Variance (ANOVA or ANOVA on Ranks) and *post hoc* tests (Holm-Sidak or Tukey) were used to determine significance (*P* ≤ 0.05).

## Results

**FIGURE 1:**
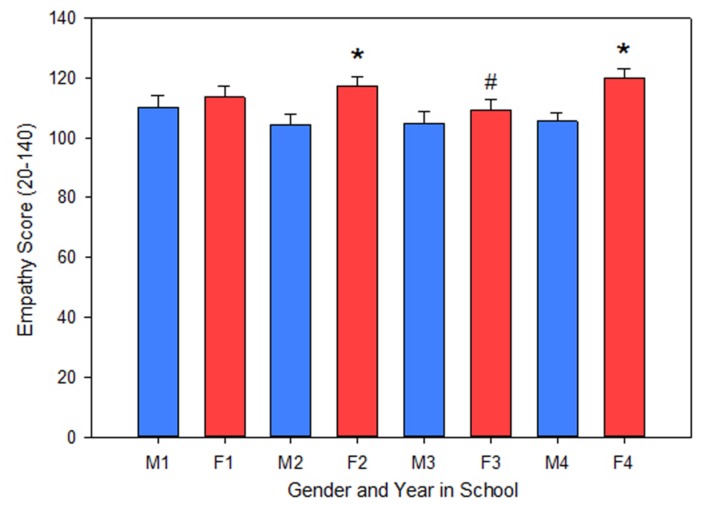
**A 2-way ANOVA was used to examine the effect of gender and class year on the Jefferson Empathy score.** Females, as an overall group, scored higher than male students (*P* < 0.001). By class year, females scored higher than males in the 2nd and 4th class years (*P* < 0.05). Additionally, 3rd year female empathy scores were significantly lower than 2nd and 4th year female scores (*P* < 0.05). M, male; F, female; 1–4, year in School; ^∗^significant difference vs. Male in class year; #significant difference vs. Female in 2nd and 4th year.

Three hundred and four pharmacy students at Midwestern University participated in the fall survey with an overall response rate of 37%. The response rate for each class year (1st to 4th) was: 31, 29, 74, and 13%, respectively. The average empathy score was 110.4 ± 0.8, on a scale of 20–140 which is comparable to empathy scores found by Fjortoft et al. (2011) and Van Winkle et al. (2012b). To compare the effect of gender and class year on the Jefferson Empathy score, a 2-way ANOVA was used. Validating prior research, females scored significantly higher than males overall (112.7 ± 0.9 vs. 106.1 ± 1.5; *P* < 0.001) and specifically in the 2nd and 4th year *P* < 0.05 (**Figure 1**). Empathy scores did not change in male students between class year, but were significantly lower in 3rd year female students vs. 2nd and 4th year female students *P* < 0.05 (**Figure 1**). There was no effect of age (*P* = 0.67), ethnicity (*P* = 0.09), religion (*P* = 0.40), marital status (*P* = 0.17), birth order (*P* = 0.22), highest family education (*P* = 0.53), or debt (*P* = 0.68) on Jefferson empathy scores.

**FIGURE 2:**
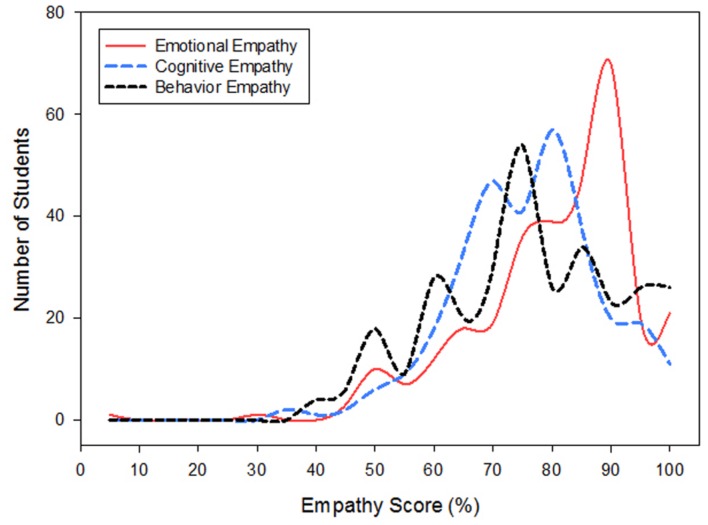
**Emotional empathy was greater than Cognitive (*P* = 0.005) and Behavioral empathy (*P* = 0.01) in Pharmacy students.** Scores and population distributions are shown.

Empathy questions were sub-divided into emotional, cognitive, and behavioral categories and answers were scored and ranked on a scale of 0–100%. To compare the effect of gender, year in pharmacy school, and empathy sub-components (behavioral, cognitive, and emotional), a 3-way ANOVA was used. As a graduate population, pharmacy students scored significantly higher in emotional empathy 78.1 ± 0.8% vs. cognitive empathy 73.9 ± 0.7% (*P* = 0.005) as well as behavioral empathy 73.8 ± 0.9% (*P* = 0.011). The distribution of emotional, cognitive, and behavioral empathy scores and student numbers are presented in **Figure 2.** There was not a significant interaction between individual subcomponents of empathy and class year (*P* = 0.441) or gender (*P* = 0.441). However, subcomponent scores were significantly lower in the 3rd year class (*P* < 0.05); **Table 1.** Similar to the Jefferson empathy scores, females scored significantly higher than males in the 2nd, 3rd, and 4th class years (*P* < 0.05); and 3rd year females scores were lower than 1st, 2nd, and 4th year female scores (*P* < 0.05); **Table 1.** Additionally, female 4th year empathy scores were greater than 1st year female scores (*P* = 0.02; **Table 1**). In male students, empathy scores were greater in 1st year vs. 3rd year (*P* = 0.024; **Table 1**).

**Table 1:** Empathy subcomponent scores %, gender, and class year are presented.

	M1	F1	M2	F2	M3	F3	M4	F4
Behavior (%)	75.2 ± 2.5	78.0 ± 2.2	65.2 ± 4.4	80.5 ± 2.1	67.1 ± 2.0	71.7 ± 1.5	72.1 ± 3.0	85.3 ± 2.7
Cognitive (%)	72.3 ± 3.0	75.4 ± 1.9	69.8 ± 3.0	79.2 ± 1.3	70.8 ± 1.8	73.3 ± 1.2	70.9 ± 3.1	78.9 ± 2.6
Emotional (%)	78.8 ± 2.8	81.0 ± 1.8	73.8 ± 3.4	83.9 ± 1.3	72.5 ± 2.4	76.9 ± 1.4	71.0 ± 2.7	87.6 ± 1.8


*M, male; F, female; 1–4, year in School. Student scores for Emotional empathy were significantly greater than Cognitive empathy (*P* = 0.005) as well as Behavioral empathy (*P* = 0.011). Third year subcomponent empathy scores were significantly lower vs. year 1 (*P* < 0.001), year 2 (*P* = 0.048), and year 4 (*P* = 0.004). Overall, females scored higher than males (*P* < 0.001; Year 1: *P* = 0.147; Year 2: *P* < 0.001; Year 3: *P* = 0.003; Year 4: *P* < 0.001). First year male students had a higher overall empathy score than 3rd year male students (*P* = 0.024). Third year female students had a lower overall empathy score than female 1st, 2nd, and 4th year students (*P* < 0.01). Additionally, female 4th year scores were significantly higher than 1st year female students (*P* = 0.023).*

**FIGURE 3:**
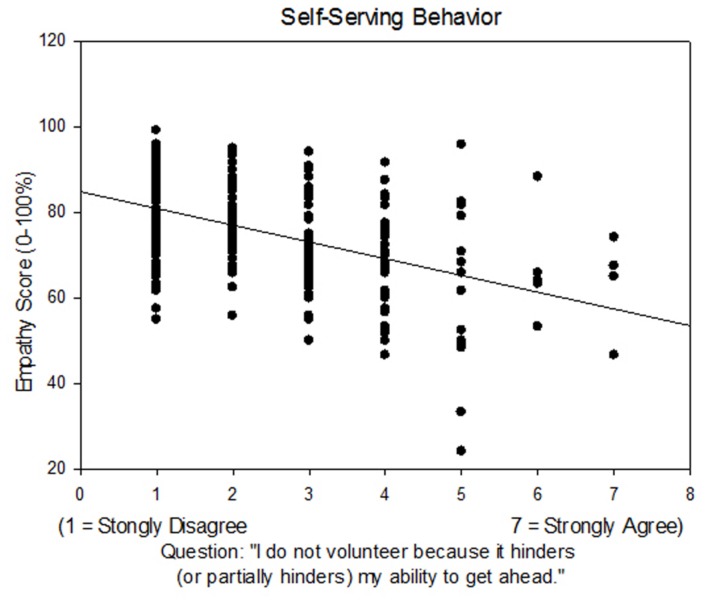
**Jefferson empathy scores were correlated to Likert scaled responses to the question, “I do not volunteer because it hinders (or partially hinders) my ability to get ahead.”** Linear regression: *R* = 0.490, *P* < 0.001.

Questions targeting personality traits such as self-serving motive, coercion, medical authoritarianism, elitism, and egalitarianism were correlated to empathy scores. The self-serving statement, “I do not volunteer because it hinders (or partially hinders) my ability to get ahead.” was negatively correlated to empathy (*R* = 0.49, *P* < 0.001) and the behavioral subcomponent of empathy (*R* = 0.371, *P* < 0.001; **Figure 3**, **Table 2**). The experience of coercion to enter a health related profession was assessed with the statement “I feel pressured to enter the health professional field.” Answers to this statement were negatively correlated to empathy (*R* = 0.344, *P* < 0.001) and closely related to the emotional subcomponent of empathy (*R* = 0.334, *P* < 0.001, **Table 2**). Medical authoritarianism was assessed by responses to “Conscientious patients deserve better health care than those with self-inflicted conditions.” and negatively correlated to empathy (*R* = 0.428, *P* < 0.001). This question had a strong cognitive empathy component (*R* = 0.396, *P* < 0.001, **Table 2**). There was a negative association between empathy scores and elitism as assessed by the question “Those who contribute the most to society should get better health care” (*R* = 0.426, *P* < 0.001). On the other hand, egalitarianism, assessed by the question, “We should do what we can to equalize health care for different groups,” was positively associated with empathy scores (*R* = 0.29, *P* < 0.001) with strong associations to behavioral empathy (*R* = 0.265, *P* < 0.001), cognitive empathy (*R* = 0.283, *P* < 0.001), and emotional empathy (*R* = 0.212, *P* < 0.001).

**Table 2:** The Jefferson empathy score and subcomponents of empathy (behavioral, cognitive, and emotional) were correlated to responses from questions that targeted self-serving motivation, medical authoritarianism, and coercion.

	Self-serving motive (behavioral)	Medical authoritarianism (cognitive)	Coercion (emotional)
Jefferson: empathy	*R* = 0.490; *P* < 0.001	*R* = 0.428; *P* < 0.001	*R* = 0.344; *P* < 0.001
Behavioral empathy	*R* = 0.371; *P* < 0.001	*R* = 0.335; *P* < 0.001	*R* = 0.211; *P* < 0.001
Cognitive empathy	*R* = 0.442; *P* < 0.001	*R* = 0.396; *P* < 0.001	*R* = 0.321; *P* < 0.001
Emotional empathy	*R* = 0.447; *P* < 0.001	*R* = 0.373; *P* < 0.001	*R* = 0.334; *P* < 0.001


*Answers to each question were negatively correlated with empathy and subcomponents. For behavioral empathy correlations, the highest R-value was associated with the question targeting a behavioral component. Correlations to cognitive and emotional empathy were greatest in the questions emphasizing cognitive and emotional aspects, respectively.*

The categorization of the 20 Jefferson empathy questions into subcomponents of empathy were not equally distributed (Behavioral 20%, Cognitive 45%, and Emotional 35%). Therefore, correlations to behavioral empathy had less power because fewer Jefferson empathy questions are related to behavior. Nonetheless, questions that targeted subcomponents of empathy were effective. Of three questions targeting behavior, cognition, or emotion; the *R*-value for behavioral empathy was greatest for the question targeting behavior (**Table 2**). The question targeting cognition had the highest *R*-value for cognitive empathy, and the question targeting emotion had the highest *R*-value for emotional empathy.

## Discussion

To the best of our knowledge, no psychometrically sound research instrument is available to measure empathy specifically among pharmacy students. Consistent with this, researchers continue to identify a significant need to develop instruments to measure empathy (Hojat et al., 2009). The Jefferson scale for empathy possesses strong psychometric properties (Hojat, 2007), including construct validity (Hojat et al., 2001, 2002b) criterion validity (Hojat et al., 2002a), test–retest reliability, and internal consistency (Hojat et al., 2001, 2002b). The Jefferson scale is also amenable to word adaptations to match the student audience, while still retaining such properties (Hojat et al., 2009). Accordingly, we adapted the Jefferson Scale of Physician Empathy to pharmacists-in-training and identified questions within the survey that target empathy subcomponents.

The present study assessed three subcomponents of empathy and how they relate to various personality and psychosocial traits in pharmacy students. Our study demonstrated that, as a graduate population, emotional empathy scored highest while overall empathy scores were lower in the 3rd year class. The observed decline in graduate health care student empathy supports prior research in nursing students (Wilson et al., 2012) as well as in medical students (Neumann et al., 2011); however, it contradicts previous data for pharmacy students (Wilson et al., 2012). One important implication of the differences observed between emotional and cognitive empathy relates to how amenable these characteristics are to change. Based on the neurological underpinning, it can be assumed that emotional empathy is less amenable to change, as it is the most primitive and elicits automatic neural processes (de Waal, 2008; Rizzolatti et al., 2009). In contrast, cognitive empathy may be substantially enhanced by education, which is supported by a body of research on controlled neural processes and cortical-based plasticity. That is, as students gain academic experience and work with patients, they develop constructs that alter their empathetic system that may either increase or decrease their empathetic response. This neurological distinction leads most investigators to hypothesize that cognitive empathy can be augmented while emotional empathy is less likely to change with time and experience.

Our additional questions revealed three personal(ity) characteristics (coercion, self-serving behavior, and medical authoritarianism) that significantly correlated with empathy scores. These characteristics were selected for two reasons. First, each characteristic independently relates to different aspects of empathy: emotional, behavioral, and cognitive, respectively. Second, these characteristics are a product of life experience, and thus may still be responsive to education and intervention programs.

Students who felt coerced to enter a health professional field demonstrated lower emotional empathy. At the same time, there was a decline in emotional empathy in 3rd vs. 2nd year students. This contradicts the theory that emotional empathy is highly embedded in our neuronal construct and less likely to change. These results suggest emotional empathy levels could be changed in the academic setting. Secondly, the data indicate that coerced students may be less equipped for pharmacy because of its consequential association with lower emotional empathy. Potential mechanisms linking coercion to depressed emotional empathy levels may be increased stress, anxiety, and general unhappiness in their chosen career. Taken together, coercion may be a dispositional factor that moderates responsiveness to educational programs, meaning that students who were coerced into the field may learn from, and benefit differently, than those who were not coerced.

In the present study, self-serving behavior was negatively correlated with behavioral empathy, as suggested by responses to a question on volunteerism. By definition, individuals who are self-serving inherently have less comfort with, and preference against, extending themselves to outreach programs. Volunteering activities may be particularly stressful because they engage the student in a process that is contrary to their dispositional tendency. This dispositional discomfort may be compounded by the fact that volunteering competes with the students’ studies for limited time, effort, and attention. Therefore, academic requirements that promote volunteering as a method to enhance empathy levels may cause more anxiety in students with a low reservoir of behavioral empathy.

Students who endorsed statements of medical authoritarianism scored lower on cognitive empathy. Recent studies have confirmed that elitist attitudes predict lower empathy and patient-centeredness^32,33^. Doctoral training explains the complex pathophysiology of and risk behaviors causing disease. Thus, treatment is simplified to pharmaceutical and behavioral modification. However, patient compliance is often substantially hindered by a myriad of psychological, social and educational barriers. Without a solid appreciation for this multilayered etiology, it becomes increasingly difficult to cognitively empathize with patients. Furthermore, studies demonstrate that medical authoritarianism increases over the course of graduate training, while empathy decays (Tsimtsiou et al., 2007). This pattern of heightened medical authoritarianism and decaying empathy may be changed by encouraging circular activities that offset other experiential factors that contribute to the decline in empathy during graduate training (Van Winkle et al., 2013b).

### Educational Implications

Educational reform that prevents the decline in empathy and embraces measureable outcomes should be considered a mandate in pharmacy training. In an effort to advance this mission, we set out to identify the relationship between individual traits and the subcomponents of empathy. In identifying these relationships, we are better positioned to target specific characteristics that either enhance or impinge upon students’ development and maintenance of empathy.

The present study demonstrated that emotional empathy was greater than cognitive or behavioral empathy. Given the relationship between excessive emotional empathy and negative clinical outcomes, it may be important for educational programs to intervene to moderate this trait, while concurrently working to enhance cognitive empathy. Whilst workshops lay the foundation and keep empathy afloat in primary years (Van Winkle et al., 2009, 2012b), a cynical transformation appears to creep in during latter years. This escalation of cynicism has long been recognized in neighboring healthcare professions (Becker, 1961; Wolf et al., 1989) and has been described as “traumatic deidealization” (Kay, 1990) and “dehumanization.” (Hojat et al., 2005). Explanatory models for this decline, may be: the development of biases against some patient populations, frustration with the health care system, or simply exhaustion from academic workload. As an extension of Midwestern University’s previous initiative, buffer programs in the 3rd year may refresh students’ cognitive empathetic abilities. Finally, a concerted effort to facilitate volunteerism and foster responsiveness to patient needs, may prevent declines in behavioral empathy (Van Winkle et al., 2013b).

### Implications for Inter-Professional Healthcare Collaborations

A prominent feature of effective healthcare providers is interdisciplinary collaboration. Collaborative models are demonstrated when traditional disciplinary boundaries are crossed through a pooling of information. Inter-professional collaboration has seen growing support in the empirical literature for its use in addressing multi-faceted problems and for enhancing therapeutic outcomes.

There are successful examples of interdisciplinary learning that result in improved collaborative scores. For example, Van Winkle et al. (2012a, 2013a) have promoted inter-disciplinary educational programs to enhance cognitive empathy and to improve collaborative scores between pharmacy and medical students. Apart from demonstrating the interdependency of cognitive empathy and collaborative attitudes, their findings also demonstrate that pharmacy students have significantly greater physician-pharmacist collaborative tendencies. In this regard, pharmacy students could favorably influence medical students toward collaboration in critical thinking/reflection exercises (Van Winkle et al., 2013a).

The academic setting is an ideal environment to foster empathy levels in graduate students pursuing health related professions. Early implementation in preclinical years may be the most effective implementation strategy, as these students are a captive audience wherein mandatory group workshops could be controlled and optimally delivered.

### Study Limitations

This exploratory survey does not have longitudinal data. Therefore, changes in class year may be due to a unique class population and/or the time course of pharmaceutical graduate education. Survey data are unique to Midwestern University and may not be reflective of all pharmacy students. Future, longitudinal studies will provide repeated measures and greater population numbers adding to the confidence of future results and validation of their educational implications.

## Conclusion

This exploratory survey of empathy in pharmacy students validates prior literature demonstrating that empathy levels are higher in females and frequently decline during the 3rd year of graduate training. Our data suggest that emotional empathy may play a greater role than cognitive and behavioral empathy in this group of students, and may be amenable to change. Academic programs could be implemented to promote volunteerism, and activities that foster awareness and responsiveness to patient needs; thereby augmenting cognitive and behavioral empathy levels and preparing students to be more effective pharmacists.

## Author Contributions

KH, MR, and CT were present at the inception of the research idea, contributed to the literature review, the IRB application and approval process, assisted in data collection and interpretation, writing of the manuscript and editing, and can defend the research in public.

## Conflict of Interest Statement

The authors declare that the research was conducted in the absence of any commercial or financial relationships that could be construed as a potential conflict of interest.

The reviewer AM declared a shared affiliation, though no other collaboration, with the authors to the handling Editor, who ensured that the process nevertheless met the standards of a fair and objective review.
