# Supplementary material for: Cognitive, Behavioral and Emotional Empathy in Pharmacy Students: Targeting Programs for Curriculum Modification
Source: Front Pharmacol. 2016 Apr 19;7:96. doi: 10.3389/fphar.2016.00096 (PMC4835498; doi:10.3389/fphar.2016.00096)
Supplement: Supplementary file 1 [file Table_1.PDF]

Jefferson Scale of Empathy (Medical Student version):

#	Question	Empathy Subcomponent
1	Physicians' understanding of their patients'…	Cognitive
2	Patients feel better when…	Emotional
3	It is difficult for a physician...	Cognitive
4	Understanding body language is...	Cognitive
5	A physician's sense of humor…	Behavioral
6	Because people are different...	Cognitive
7	Attention to patients' emotions...	Emotional
8	Attentiveness to patients' personal…	Cognitive
9	Physicians should try to…	Cognitive
10	Patients value a physician's…	Emotional
11	Patients' illnesses can be cured…	Emotional
12	Asking patients about…	Behavioral
13	Physicians should try to…	Cognitive
14	I believe that emotion…	Emotional
15	Empathy is a therapeutic…	Behavioral
16	Physicians' understanding of the…	Emotional
17	Physicians should try to…	Cognitive
18	Physicians should not allow…	Emotional
19	I do not enjoy…	Behavioral
20	I believe that…	Cognitive

Appendix 1: The nature of each question in the Jefferson Scale of Empathy (Medical Student version) is listed with our classification scheme of empathy subcomponents. © *Thomas Jefferson University, 2001. All rights reserved.* Jefferson, as the sole copyright holder, maintains the copyright for granting or declining permission for any additional use of any and all versions of the JSE.
